# The Predictors of Early Treatment Effectiveness of Intravitreal Bevacizumab Application in Patients with Diabetic Macular Edema

**DOI:** 10.3390/diagnostics14100992

**Published:** 2024-05-10

**Authors:** Karla Katić, Josip Katić, Marko Kumrić, Joško Božić, Leida Tandara, Daniela Šupe Domić, Kajo Bućan

**Affiliations:** 1 Department of Ophthalmology, University Hospital of Split, 21000 Split, Croatia; 2 Department of Cardiology, University Hospital of Split, 21000 Split, Croatia; josipkati@gmail.com; 3 Department of Pathophysiology, University of Split School of Medicine, 21000 Split, Croatia; kumricjudo@gmail.com (M.K.); josko.bozic@mefst.hr (J.B.); 4 Department of Medical Laboratory Diagnostics, University Hospital of Split, 21000 Split, Croatia; leida.tandara@gmail.com (L.T.); daniela.supedomic@gmail.com (D.Š.D.); 5 Department of Medical Chemistry and Biochemistry, University of Split School of Medicine, 21000 Split, Croatia; 6 University Department of Health Studies, University of Split, 21000 Split, Croatia; 7 Department of Ophthalmology, University of Split School of Medicine, 21000 Split, Croatia

**Keywords:** diabetic macular edema (DME), non-proliferative diabetic retinopathy (NPDR), bevacizumab, neutrophil-to-lymphocyte ratio (NLR), monocyte-to-lymphocyte ratio (MLR), platelet-to-lymphocyte ratio (PLR), systemic immune-inflammation index (SII), apolipoprotein B to A-I ratio (ApoB/ApoA-I), vitamin D

## Abstract

The aim of this study was to establish whether multiple blood parameters might predict an early treatment response to intravitreal bevacizumab injections in patients with diabetic macular edema (DME). Seventy-eight patients with non-proliferative diabetic retinopathy (NPDR) and DME were included. The treatment response was evaluated with central macular thickness decrease and best corrected visual acuity increase one month after the last bevacizumab injection. Parameters of interest were the neutrophil-to-lymphocyte ratio (NLR), monocyte-to-lymphocyte ratio (MLR), platelet-to-lymphocyte ratio (PLR), systemic immune-inflammation index (SII), vitamin D, and apolipoprotein B to A-I ratio (ApoB/ApoA-I). The NLR (2.03 ± 0.70 vs. 2.80 ± 1.08; *p* < 0.001), MLR (0.23 ± 0.06 vs. 0.28 ± 0.10; *p* = 0.011), PLR (107.4 ± 37.3 vs. 135.8 ± 58.0; *p* = 0.013), and SII (445.3 ± 166.3 vs. 675.3 ± 334.0; *p* < 0.001) were significantly different between responder and non-responder groups. Receiver operator characteristics analysis showed the NLR (AUC 0.778; 95% CI 0.669–0.864), PLR (AUC 0.628; 95% CI 0.511–0.735), MLR (AUC 0.653; 95% CI 0.536–0.757), and SII (AUC 0.709; 95% CI 0.595–0.806) could be predictors of response to bevacizumab in patients with DME and NPDR. Patients with severe NPDR had a significantly higher ApoB/ApoA-I ratio (0.70 (0.57–0.87) vs. 0.61 (0.49–0.72), *p* = 0.049) and lower vitamin D (52.45 (43.10–70.60) ng/mL vs. 40.05 (25.95–55.30) ng/mL, *p* = 0.025). Alterations in the NLR, PLR, MLR, and SII seem to provide prognostic information regarding the response to bevacizumab in patients with DME, whilst vitamin D deficiency and the ApoB/ApoA-I ratio could contribute to better staging.

## 1. Introduction

The most prevalent microvascular complication of diabetes mellitus, i.e., diabetic retinopathy (DR), is the main cause of vision loss worldwide, affecting one in four people with type 2 diabetes mellitus (T2DM) [1]. However, diabetic macular edema (DME), which can occur at any stage of DR, is the primary cause of central visual impairment in the diabetic population [2]. DME implies the accumulation of excess fluid in the macular region within the retina, typically in the inner nuclear and outer plexiform layer, Henle’s fiber layer, and subretinal space [2]. DR is a multifactorial disorder that comprises hyperglycemia, chronic inflammation, vascular permeability, retinal ischemia, angiogenesis, and neurodegeneration [3]. Chronic inflammation at any stage of DR leads to increased local and systemic levels of inflammatory molecules, including cell adhesion molecules, vascular endothelial growth factor, chemokines, and cytokines, that result in the breakdown of the blood–retinal barrier and formation of hemorrhages, exudations, and retinal edema [4]. A variety of blood count parameters, such as the neutrophil-to-lymphocyte ratio (NLR), monocyte-to-lymphocyte ratio (MLR), platelet-to-lymphocyte ratio (PLR), systemic immune-inflammation index (SII), vitamin D, and apolipoproteins A-I and B (ApoA-I and ApoB) have been established as (anti)inflammatory biomarkers related to DR and DME [5,6,7,8]. Anti-vascular endothelial growth factor (anti-VEGF) injections have recently become the standard treatment for patients with DME [9]. Bevacizumab is a humanized monoclonal antibody that inhibits all isoforms of vascular endothelial growth factor A [10]. It is still commonly utilized as an off-label therapy for DME [11]. Although significant treatment success of DME has been established with bevacizumab, a subset of patients appears refractory and often needs alternative anti-VEGF therapies and/or steroids for medical management [9,10,11].

The principal aim of this study was to establish whether the aforementioned blood parameters can predict an early treatment response to intravitreal injections of bevacizumab in patients with DME and non-proliferative diabetic retinopathy (NPDR). The additional aim was to establish an association between the severity of NPDR and various clinical and laboratory parameters.

## 2. Materials and Methods

This prospective observational study was conducted at the Department of Ophthalmology, University Hospital Centre Split in Croatia from March 2022 to March 2023. Informed written consent was obtained from each participant enrolled in the study, while the study protocol was approved by the Ethics Committee of the University Hospital Centre Split (Approval No. 2181-147/01/06/M.S.-22-03) and conducted in accordance with all ethical principles of the Declaration of Helsinki. 

Patients aged 18 years and older suffering from T2DM, NPDR, and DME were included in this research. Exclusion criteria were vitreous opacities and cataracts that reduce the visibility of the ocular fundus, eye surgery within 6 months, previous vitreoretinal surgery and laser photocoagulation therapy, patients with silicone oil in the eye, proliferative DR, epiretinal membrane or vitreoretinal traction, acute conjunctivitis or blepharitis, glaucoma, and uveitis. Patients with systolic blood pressure > 180 mmHg or diastolic blood pressure > 110 mmHg, any thromboembolic event within 3 months, and those using substitutional vitamin D therapy and systemic steroids were also excluded from the present study. Finally, patients receiving intraocular anti-VEGF therapy and periocular or intravitreal steroids within the last 3 months were excluded as well. 

Before applying the first intravitreal injection, all patients had their medical history reviewed and underwent a detailed ophthalmological examination by the same ophthalmologist, including fundus examination and optical coherence tomography. Patients also underwent standard physical examination. Body weight and height were measured using a scale with an integrated altitude meter (Seca, Birmingham, UK). Their body mass index was calculated by dividing the value of body mass (kg) and squared height (m^2^). 

Peripheral blood sampling was performed in the fasting state by an experienced nurse prior to the first bevacizumab injection. A maximum of 22 mL of blood was drawn from the cubital vein, and samples were analyzed using standard operating procedures in the same certified institutional biochemical laboratory. The biochemist who performed the analysis was blinded to the participant’s assignment to the group. All the serum parameters were determined by an automatic biochemical analyzer (Beckman Coulter Inc., Miami, FL, USA) immunoturbidimetrically with appropriate kits at the Department of Medical Laboratory Diagnostics, University Hospital of Split. The SII was calculated by the formula: neutrophil × platelet/lymphocyte.

All patients were diagnosed and treated as per the current guidelines for the diagnosis and treatment of DME [12]. The severity of DR was classified using the International Clinical Diabetic Retinopathy and Diabetic Macular Edema Disease Severity Scores [13]. Best corrected visual acuity (BCVA) was measured based on the Snellen chart and recorded as the logarithm of the minimum angle of resolution (logMAR). The optical coherence tomography (OCT) was performed using CIRRUS^®^ 6000 OCT (Carl Zeiss^®^, Oberkochen, Germany), to confirm the presence of DME and to measure the baseline central macular thickness (CMT) before applying the first intravitreal injection of bevacizumab. Three monthly injections of bevacizumab (Avastin^®^; Genentech, South San Francisco, CA, USA) 1.25 mg/0.05 mL were given to all patients. Therapeutic success was measured by the resolution of a minimal 10% of macular thickness on OCT and improvement of VA for one or more lines on the Snellen chart four weeks after the last intravitreal injection. For those patients with bilateral DME, the eye that responded better to the treatment was selected to be enrolled in this study. 

### Statistical Analysis

MedCalc Statistical Software version 20.113 (MedCalc Software BV, Ostend, Belgium) and GraphPad Prism version 9.4.1 for Windows (GraphPad, La Jolla, CA, USA) were used in this study. Quantitative data were presented as mean ± standard deviation or median and interquartile range, depending on data distribution, whereas qualitative data were expressed as a number and percentage. The Shapiro–Wilk test was used to assess the normality of data distribution. Qualitative variables were compared using the Chi-squared test. Normally distributed data were compared using Student’s *t*-test, unlike non-normally distributed data, which were compared using the Mann–Whitney U test. The correlation between parameters of interest has been examined using Spearman’s rank correlation coefficient. The accuracy of multiple parameters (NLR, MLR, PLR, and SII) in predicting response to anti-VEGF was assessed using receiver operator characteristics (ROC) analysis, with a calculation of area under the curve (AUC). Furthermore, to establish whether the association between the above-noted parameters and response to anti-VEGF therapy is independent of age, sex, disease duration, and C-reactive protein, we employed multiple logistic regression analysis. Accordingly, multiple linear regression analysis was used to determine the relative contribution of independent variables in the prediction of reduction in CMT after therapy. For the detection of multicollinearity in the linear regression analysis, the variance inflation factor (VIF) was used. Statistical significance was *p* < 0.05 for all comparisons.

## 3. Results

The baseline characteristics of patients are presented in Table 1. A total of 78 patients were included in the present study. Female sex and hypertension were more prevalent in the non-responder group (*p* = 0.044 and *p* = 0.006, respectively). The NLR (2.03 ± 0.70 vs. 2.80 ± 1.08; *p* < 0.001), MLR (0.23 ± 0.06 vs. 0.28 ± 0.10; *p* = 0.011), PLR (107.4 ± 37.3 vs. 135.8 ± 58.0; *p* = 0.013), and SII (445.3 ± 166.3 vs. 675.3 ± 334.0; *p* < 0.001) were significantly different between the responder and non-responder groups. Apart from various immune cell ratios (NLR, MLR, PLR, and SII), in Table 2 it is apparent the groups were different in regards to baseline CMT (*p* = 0.032), BCVA (0.020), and the number of previous anti-VEGF injections (*p* = 0.038). 

**Figure 1:**
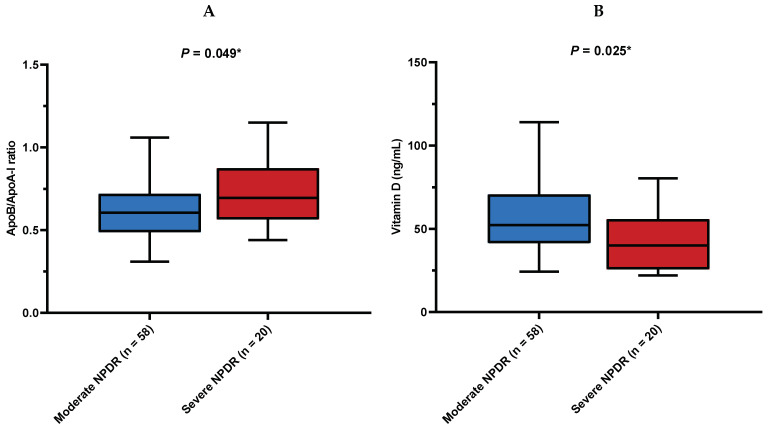
(**A**) Comparison of the ApoB/ApoA-I ratio between stages of NPDR. (**B**) Comparison of vitamin D concentrations between stages of NPDR. ApoB/ApoA-I ratio = apolipoprotein B to apolipoprotein A-I ratio; NPDR = non-proliferative diabetic retinopathy. Data is presented as median (IQR). * Mann–Whitney U test.

**Figure 2:**
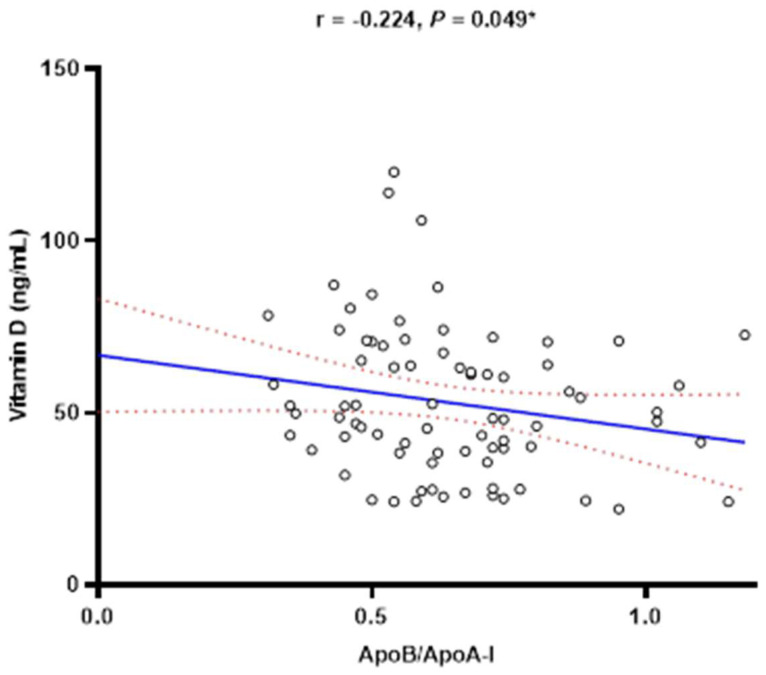
Correlation between vitamin D serum concentrations and the ApoB/ApoA-I ratio. ApoB/ApoA-I ratio = apolipoprotein B to apolipoprotein A-I ratio. * Spearman’s rank correlation coefficient.

Patients with the severe stage of NPDR had a significantly higher ApoB/ApoA-I ratio when compared to patients with moderate NPDR (0.70 (0.57–0.87) vs. 0.61 (0.49–0.72), *p* = 0.049) (Figure 1A). On the other hand, patients with the severe stage of NPDR had significantly lower vitamin D serum concentrations in comparison to patients with moderate NPDR (52.45 (43.10–70.60) ng/mL vs. 40.05 (25.95–55.30) ng/mL, *p* = 0.025) (Figure 1B). Accordingly, a weak negative correlation has been observed between serum vitamin D concentration and the ApoB/ApoA-I ratio (r = −0.224, *p* = 0.049) (Figure 2). Differences in various variables of interest between different stages of NPDR are presented in Supplementary Table S1. There was no significant difference in the NLR, MLR, PLR, and SII regarding the severity of NPDR.

Multivariable linear regression analyses demonstrated that the NLR, PLR, MLR, and SII are all independent predictors of change in CMT following bevacizumab injections, irrespective of age, sex, disease duration, and C-reactive protein (Table 3).

**Figure 3:**
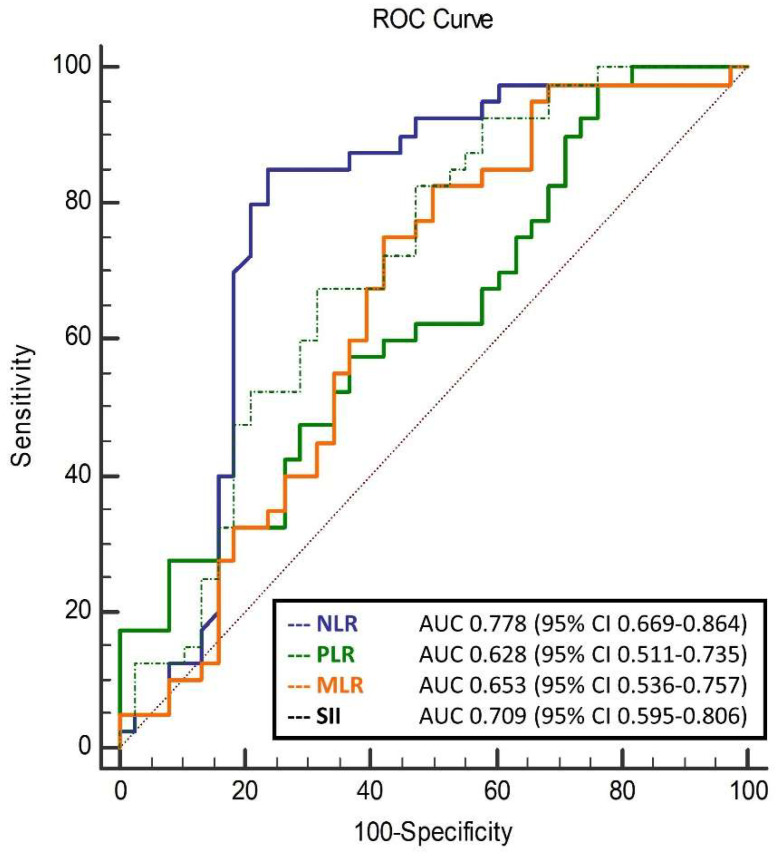
Receiver operator characteristics analysis of selected predictors for response to intravitreal anti-VEGF therapy in patients with NPDR. NPDR = non-proliferative diabetic retinopathy; NLR = neutrophil-to-lymphocyte ratio; MLR = monocyte-to-lymphocyte ratio; PLR = platelet-to-lymphocyte ratio; SII = systemic immune-inflammation index; AUC = area under the curve; and CI = confidence interval.

In the ROC analysis, the NLR, PLR, MLR, and SII were all shown to be predictors of response to bevacizumab therapy in patients with NPDR (Figure 3). Among them, the NLR was shown to have the biggest AUC (0.778 (95% CI 0.669–0.864), *p* < 0.001), i.e., the best accuracy in predicting response to bevacizumab. Moreover, it was confirmed in multivariable logistic regression that all of the above ratios remain significant after adjustment for age, sex, disease duration, and C-reactive protein (Table 4). Correlations between change in CMT and selected biomarkers are presented in Supplementary Table S2. Significant negative correlations were noted for baseline CMT (r = −0.310; *p* = 0.001) and baseline BCVA (logMAR) (r = −0.224; *p* = 0.031).

## 4. Discussion

Our study revealed that among patients who exhibited a significant improvement in CMT, there were distinct differences in multiple biomarkers when compared to those who did not respond to the intravitreal bevacizumab therapy. Specifically, the NLR, MLR, PLR, and SII were shown to predict the response to anti-VEGF therapy, independent of age, sex, disease duration, and C-reactive protein levels. Accordingly, the NLR, PLR, and MLR were shown to predict the reduction in CMT following bevacizumab injections independently of the above-noted factors. In the present study, we demonstrated that patients with the severe form of NPDR showed a significantly higher ApoB/ApoA-I ratio and lower HDL-C/ApoB ratio, along with lower levels of vitamin D, in comparison to moderate NPDR. Finally, the study showed that patients who responded to the therapy had worse baseline BCVA and CMT compared to those who did not respond. 

In recent times, there has been a discovery of systemic inflammatory markers, such as white blood cells (WBCs) and platelet counts, together with their ratios, which serve as diagnostic indicators for understanding the development of DME [14]. The involvement of inflammatory, angiogenic, and oxidative stress pathways may potentially contribute to the phenomenon of leukocyte adherence to the endothelial cell wall, with a particular emphasis on neutrophils and monocytes [15]. These pathological processes can result in the depletion of pericytes, heightened vascular permeability, disruption of the blood–retinal barrier, and ultimately contribute to the development of DME by promoting the augmented release of VEGF [15]. Therefore, the significance of anti-VEGF medications becomes apparent in the management of DME [16]. In recent years, a limited number of studies have been conducted to examine the impact of systemic inflammatory biomarkers on DME and the response to intravitreal therapy with anti-VEGF agents [17,18,19]. Inflammatory biomarkers related to WBCs, including the NLR, PLR, MLR, and SII can be easily obtained from the complete blood cell count. These markers have been found to exhibit associations with diabetes and its accompanying complications [17]. The adhesion of neutrophils to the endothelial cell wall can lead to the advancement of inflammation and microangiopathy. The stability of the NLR surpasses that of individual blood neutrophils and lymphocytes due to it having less susceptibility to various physiological and pathological situations [17]. This suggests that modifying the NLR may provide a more accurate representation of the inflammatory state and immune responses. The research yielded statistically significant findings, indicating a positive connection between an NLR greater than or equal to 2 and a monocyte-to-high density lipoprotein cholesterol ratio (MHR) more than or equal to 13.9 in predicting the occurrence of DME in individuals aged 64 and above [17]. Nevertheless, there were no significant correlations observed between an NLR ≥ 2 or an MHR > 13.9 and outcomes pertaining to central retinal thickness or BCVA subsequent to anti-VEGF treatment. It is worth noting that there was a notable correlation between an elevated NLR and unfavorable results in terms of central retinal thickness [17]. Hu et al. conducted a study with the objective of evaluating the prognostic value of the NLR in persons receiving monthly ranibizumab treatment for DME [18]. Patients who had an NLR below 2.27 had more significant enhancements in BCVA in comparison to those whose NLR values were over 2.27 [18]. This finding suggests that a pretreatment high NLR is a reliable prognostic factor, significantly associated with less favorable improvements in BCVA among patients with DME who are undergoing intravitreal ranibizumab therapy [18]. In a study conducted by Karimi and colleagues, it was shown that patients with DME who received intravitreal bevacizumab treatment for a duration of three months exhibited improvements in BCVA and CMT [19]. These improvements were observed to be more pronounced in individuals who saw an increase in their lymphocyte count and a decrease in their neutrophil count [19]. Furthermore, an additional exploratory analysis in our study revealed that the NLR was significantly lower and independently positively correlated with the defined treatment response when considering a response to therapy. Based on our findings, it can be concluded that the NLR demonstrated a high level of sensitivity and specificity in predicting responses to anti-VEGF therapy. In other words, the absence of a low NLR was significantly associated with a higher risk of treatment failure following intravitreal injections of bevacizumab. 

In contrast, monocytes and platelets play a crucial role in the production and secretion of proinflammatory cytokines, prooxidant cytokines, and adhesion molecules [20]. There are only a few studies that analyzed the value of an MLR in relation to DR so far, but this is the first investigation of the correlation between a PLR and MLR with treatment responses. The results of this study suggest that the PLR and MLR are independent predictors of the response to anti-VEGF therapy. Finally, ROC curve analysis demonstrated that the PLR and MLR provide significant diagnostic efficacy in distinguishing patients with a positive response to the administered therapy. These markers could thus serve as surrogate indicators in screening patients who might have a favorable response to the bevacizumab treatment.

An additional systemic biomarker that was examined in our study was the SII. According to prior research conducted by Elbeyli et al., it was determined that individuals diagnosed with NPDR and DME exhibited significantly elevated SII values in comparison to those without DME [21]. The findings strongly indicate a significant association between an increased SII value and the onset of DME [21]. In a study conducted by Özata Gündoğdu et al., it was further corroborated that levels of SII, as well as neutrophils and NLR, exhibited a statistically significant elevation in patients diagnosed with serous macular detachment as a component of DME [22]. To the best of our knowledge, this is the first study to examine the correlation between the SII and therapeutic response parameters within the DR population. It has been demonstrated that the SII exhibits a negative correlation with changes in BCVA and a positive correlation with CMT changes following the administered therapy, with confirmed diagnostic value through ROC analysis. The findings from our study align with existing pathophysiological mechanisms in explaining the potential positive associations of the SII, an immune-inflammatory indicator, with treatment responses. Hence, the SII could also serve as a potential marker for making treatment decisions involving anti-VEGF therapy in patients with DR. 

The NLR, MLR, PLR, and SII did not show significant differences in moderate and severe NPDR in our study, which is in line with other studies [23,24,25]. Zeng et al. showed that the NLR, PLR, and MLR were significantly higher in patients with DR compared to patients without DR; however, there was no relationship between the MLR, NLR, or PLR and severity of retinopathy [23]. According to Dascalu et al., the NLR, MLR, and SII were significantly higher in patients with proliferative diabetic retinopathy compared to those with NPDR or without DR [24]. The aforementioned biomarkers are more related to DME, which was also recently validated in the research by Yanxia et al., who found notable variations in biomarkers in various types of DME [25].

Our research was the first to demonstrate, using multivariant logistic regression, that biomarkers including the NLR, MLR, PLR, and SII, even after adjustment for sex, age, disease duration, and C-reactive protein, may be useful in improving the prediction of an early treatment response to intravitreal bevacizumab. These biomarkers may help identify more effectively individuals, in clinical practice, who will respond better to intravitreal anti-VEGF therapy so the patients can be consulted about the prognosis of the treatment.

Based on the results of this study, a higher ApoB/ApoA-I ratio and lower HDL-C/ApoB ratio significantly differentiated the severe form of NPDR from moderate NPDR. Emerging evidence suggests that hyperlipidemia is associated not only with cardiovascular disease but also with T2DM and its complications, such as DR [26]. In conjunction with hyperglycemia, hypertension, and insulin resistance, dyslipidemia causes alterations in numerous biochemical pathways and the signaling of growth factors [27]. This induces the production of reactive oxygen species and free radicals within cells [27]. As a result, this sequence of events compromises the blood–retinal barrier, causing injury to both blood vessels and neuro-glial cells. As a consequence, DR begins to progress [27]. This disruption of the blood–retinal barrier also contributes to lipid particle leakage and accumulation in the retinal tissue, which results in retinal lipotoxicity and elevated oxidative stress [28]. The HDL-C particle exhibits a crucial anti-atherosclerotic effect, and its antioxidant and anti-inflammatory properties have been shown to have beneficial effects on cardiovascular and inflammatory diseases [29]. Lower HDL-C has been implicated in the development of DR and its progression to severe stages, according to numerous studies [30,31,32]. Several apolipoproteins, including ApoA-I, apolipoprotein C3, ApoB, and apolipoprotein A5, are associated with lipid metabolism and are found in serum lipoprotein complexes. These apolipoproteins have been linked to the development and progression of DR [33]. In contrast to individuals without diabetes, patients with diabetes exhibited elevated ApoA-I expression within the retinal pigment epithelium [34]. This heightened expression potentially offers a safeguard against the harmful effects of lipid lipotoxicity and deposition [34]. Furthermore, prior findings consistently indicated an inverse relationship between the serum levels of ApoA-I and the progression of DR [35]. In contrast, ApoB has been shown to have a positive correlation with the severity of DR [36,37]. In a systematic review, Soedarman et al. concluded that apolipoprotein parameters are associated with the presence of DR, especially with more severe stages of DR, and that the ApoB/ApoA-I ratio is a more accurate predictor of DR severity than either ApoB or ApoA-I alone [33]. Analyzing the ApoB/ApoA-I ratio yielded more consistent findings than interpreting ApoA-I and ApoB results separately [8,36]. The ApoB/ApoA-I ratio reflects the balance of atherogenic and anti-atherogenic factors, and the imbalance between serum levels of ApoA-I and ApoB was more significant in the pathogenesis of DR than ApoA-I level alone [38,39]. Importantly, beyond the ApoB/ApoA-I ratio as a significant factor associated with the severity of DR as shown in our results, our novel finding that the serum HDL-C/ApoB ratio differs between DR stages further addresses the role of lipids and lipoproteins in DR progression. Therefore, the ApoB/ApoA-I and HDL-C/ApoB ratios may be useful to further stratify patients with DR in the clinical setting.

The finding that patients with severe NPDR showed significantly lower levels of vitamin D in comparison to the moderate NPDR group emphasizes the importance of managing changeable risk factors (i.e., vitamin D deficiency) in the prevention of DR progression. A diverse spectrum of effects associated with vitamin D was delineated [40]. There is compelling evidence suggesting the potential involvement of vitamin D in the etiology of DR [41]. Furthermore, individuals with a pronounced deficiency in vitamin D seem to exhibit an elevated susceptibility to the development of DR [42]. With regard to the severity of DR, individuals in more advanced disease stages manifested attenuated levels of 25-hydroxyvitamin D and a heightened incidence of vitamin D deficiency, according to recent meta-analyses [43,44]. A notable difference in mean serum vitamin D levels was observed across various stages of DR [45,46,47]. When considering the pathophysiological impact of vitamin D on the progression of DR, the mechanism that regulates angiogenesis is emphasized. It has been demonstrated that the active metabolite of vitamin D, calcitriol, exerts an inhibitory effect on angiogenesis and that such an inhibitory effect of calcitriol on VEGF is manifested through the reduction in neovascularization and macular edema in clinical practice [48,49,50]. In summary, vitamin D may play a significant role as a nutritional factor whose application can be modified, especially in patients with DR, all with the aim of providing protection and reducing the risk of advanced stages of DR. 

When interpreting the outcomes of this research, it is important to consider several limitations. The participants were exclusively drawn from a single medical institution, leading to a small sample population. Furthermore, using only one pretreatment measurement of systemic biomarkers proves inadequate for assessing treatment response effects. Also, there was an absence of a control group that did not receive anti-VEGF treatment, underscoring the potential necessity for designing a randomized control study to establish causality. In addition, some analyses reflect a single point in time, thus preventing us from making causal inferences. The study primarily examined the short-term impact of immune dynamics on anti-VEGF therapy, as the patients were tracked for a period of four months. It is important to mention a difference in the number of intravitreal injections individual patients received, as a greater number of consecutive injections might yield different results. Patients previously receiving more intravitreal anti-VEGF injections had a worse response to intravitreal bevacizumab therapy, which could also be a predictor of treatment response along with inflammation parameters.

## 5. Conclusions

In conclusion, alterations in the NLR, PLR, MLR, and SII seem to provide valuable prognostic informations regarding the response to anti-VEGF therapy in patients with DR, on top of the insights offered by established imaging modalities in clinical practice. Additionally, such results highlight the role of inflammation in DR pathophysiology. Although the role of ratios such as NLR seems promising in terms of prediction of anti-VEGF therapy response, whether these have added clinical benefit in patients with DR remains to be determined in larger cohorts. The results of the present study also imply the role of vitamin D deficiency and the ApoB/ApoA-I ratio in the progression of DR. In clinical terms, vitamin D and the ApoB/ApoA-I ratio could potentially contribute to better staging of these patients.

## Figures and Tables

**Table 1:** Baseline characteristics of the study population based on response to intravitreal bevacizumab.

Parameters	Total Population (*n* = 78)	Responders (*n* = 40)	Non-Responders (*n* = 38)	*p*-Value
Age (years)	67.2 ± 8.6	67.4 ± 7.4	67.0 ± 9.8	0.848 *
Female sex, *n* (%)	36 (46)	14 (35)	22 (58)	0.044 †
Smoking, *n* (%)	20 (26)	12 (30)	8 (21)	0.369 †
Duration of DM (years)	17.4 ± 10.1	17.6 ± 10.1	17.1 ± 10.2	0.847 *
Arterial hypertension, *n* (%)	59 (75.6)	25 (62.5)	34 (89.5)	0.006 †
Chronic renal failure, *n* (%)	18 (23.1)	9 (22.5)	9 (23.7)	0.902 †
eGFR (mL/min/1.73 m^2^)	67.9 ± 23.4	67.7 ± 23.3	68.1 ± 23.7	0.941 *
BMI (kg/m^2^)	26.9 (25.5–29.4)	26.3 (25.0–29.2)	27.6 (25.9–29.8)	0.143 ‡
HbA1c (%)	7.5 (6.6–8.4)	7.6 (6.6–8.6)	7.4 (6.4–8.0)	0.345 ‡
HDL-C (mmol/L)	1.3 (1.1–1.6)	1.3 (1.0–1.5)	1.4 (1.1–1.6)	0.609 ‡
LDL-C (mmol/L)	2.4 (1.6–3.1)	2.3 (1.5–2.9)	2.5 (1.8–3.2)	0.522 ‡
ApoA-I (g/L)	1.49 ± 0.28	1.47 ± 0.27	1.50 ± 0.29	0.609 *
ApoB (g/L)	0.93 ± 0.25	0.93 ± 0.25	0.94 ± 0.25	0.769 *
ApoB/ApoA-I	0.62 (0.50–0.74)	0.62 (0.51–0.72)	0.62 (0.48–0.74)	1.000 ‡
Neutrophils (×10^9^/L)	4.6 ± 1.2	4.2 ± 0.9	4.9 ± 1.3	0.006 *
Lymphocytes (×10^9^/L)	2.1 ± 0.6	2.2 ± 0.5	1.9 ± 0.6	0.031 *
Monocytes (×10^9^/L)	0.49 ± 0.14	0.48 ± 0.11	0.51 ± 0.17	0.466 *
Platelets (×10^9^/L)	229 (186–259)	226 (175–267)	235 (193–258)	0.628 ‡
NLR	1.96 ± 0.67	2.03 ± 0.70	2.80 ± 1.08	<0.001 *
MLR	0.22 ± 0.06	0.23 ± 0.06	0.28 ± 0.10	0.011 *
PLR	103.6 ± 37.1	107.4 ± 37.3	135.8 ± 58.0	0.013 *
SII	423.9 ± 163.9	445.3 ± 166.3	675.3 ± 334.0	<0.001 *
Monocyte/ApoA-I ratio	0.35 (0.26–0.42)	0.33 (0.26–0.41)	0.34 (0.26–0.42)	0.873 ‡
HDL/ApoB ratio	1.4 (1.1–1.9)	1.4 (1.1–1.9)	1.4 (1.1–1.9)	0.893 ‡
Vitamin D (nmol/L)	49.3 (38.8–67.5)	46.4 (27.9–63.4)	52.1 (41.4–70.6)	0.225 ‡
Therapy, *n* (%)				
Statins	49 (62.8)	26 (65.0)	23 (60.5)	0.685 †
Oral antidiabetics	65 (83.3)	32 (80.0)	33 (86.8)	0.421 †
Insulin	41 (52.6)	20 (50.0)	21 (55.3)	0.644 †
GLP-1RA	9 (11.5)	4 (10.0)	5 (13.2)	0.665 †

* Student’s *t*-test. † Chi-squared test. ‡ Mann–Whitney U test. Data expressed as mean ± standard deviation, median (interquartile range, IQR), or as number (%). eGFR = estimated glomerular filtration rate; BMI = body mass index; HbA1c = hemoglobin A1c; HDL-C = high-density lipoprotein cholesterol; LDL-C = low-density lipoprotein cholesterol; ApoA-I = apolipoprotein A-I, ApoB = apolipoprotein B; ApoB/ApoA-I = apolipoprotein B to apolipoprotein A-I ratio; NLR = neutrophil-to-lymphocyte ratio; MLR = monocyte-to-lymphocyte ratio; PLR = platelet-to-lymphocyte ratio; SII = systemic immune-inflammation index; Monocyte/ApoA-I = monocyte-to-apolipoprotein A-I ratio; HDL-C/ApoB = high-density lipoprotein cholesterol-to-apolipoprotein B ratio; and GLP-1RA = glucagon-like peptide-1 agonists.

**Table 2:** Ophthalmologic baseline characteristics of the study population based on response to intravitreal bevacizumab.

Parameters	Total Population (*n* = 78)	Responders (*n* = 40)	Non-Responders (*n* = 38)	*p*-Value
Baseline BCVA (logMAR)	0.40 (0.30–1.00)	0.56 (0.35–1.30)	0.35 (0.22–0.70)	0.020 ‡
Final BCVA (logMAR)	0.35 (0.20–0.70)	0.35 (0.18–0.65)	0.33 (0.22–0.70)	0.409 ‡
ΔBCVA (logMAR)	−0.09 (−0.18 to 0.00)	−0.18 (−0.48 to −0.10)	0.00 (−0.06 to 0.00)	<0.001 ‡
Baseline CMT (μm)	421 (352–481)	460 (370–504)	409 (350–448)	0.032 ‡
Final CMT (μm)	372 (303–430)	344 (291–391)	404 (334–450)	0.006 ‡
ΔCMT (μm)	−44 (−88 to −14)	−88 (−137 to −65)	−12 (−23 to 7)	<0.001 ‡
Pseudophakia, *n* (%)	18 (23.1)	8 (20.0)	10 (26.3)	0.511 ‡
Type of macular edema, *n* (%)				
CME	41 (52.6)	23 (57.5)	18 (47.4)	0.549 †
SLDRT	34 (43.6)	15 (37.5)	19 (50.0)	
SRF	3 (3.8)	2 (5.0)	1 (2.6)	
Number of previous anti-VEGF injections	3 (0–5)	2 (0–4)	3 (0–6)	0.038 ‡
Diabetic retinopathy severity, *n* (%)				
Moderate NPDR	58 (74.4)	33 (82.5)	25 (65.8)	0.093 †
Severe NPDR	20 (25.6)	7 (17.5)	13 (34.2)	

† Chi-squared test. ‡ Mann–Whitney U test. Data expressed as mean ± standard deviation, median (interquartile range, IQR), or as number (%). Baseline BCVA = baseline best-corrected visual acuity; final BCVA = final best-corrected visual acuity; ΔBCVA = best-corrected visual acuity change between final and baseline; baseline CMT = baseline central macular thickness; final CMT = final central macular thickness; ΔCMT = central macular thickness change between final and baseline; CME = cystoid macular edema; SLDRT = sponge-like diffuse retinal thickness; SRF = subretinal fluid; moderate NPDR = moderate non-proliferative diabetic retinopathy; and severe NPDR = severe non-proliferative diabetic retinopathy.

**Table 3:** Univariable and multivariable linear regression models that examined selected biomarkers as the predictors of change in CMT following bevacizumab therapy.

Variable	Univariable Model		Multivariable Model	
	r-Correlation Coefficient	*p*-Value	β ± SE	*p*-Value
NLR	0.48	<0.001	7.58 ± 1.59	<0.001
MLR	0.49	<0.001	98.4 ± 17.9	<0.001
PLR	0.35	0.002	0.11 ± 0.03	0.002
SII	0.47	<0.001	0.03 ± 0.01	<0.001

All multivariable linear regression analyses were tested separately for each ratio of interest (the NLR, MLR, and PLR) and adjusted for covariates of age, sex, C-reactive protein, and disease duration. CMT = central macular thickness; NLR = neutrophil-to-lymphocyte ratio; MLR = monocyte-to-lymphocyte ratio; PLR = platelet-to-lymphocyte ratio; SII = systemic immune-inflammation index; β = the standardized beta; and SE = the standard error.

**Table 4:** Multivariable logistic regression models that examined selected biomarkers as the predictors of response to anti-VEGF therapy.

Variable	Multivariable Model	
	OR (95% CI)	*p*-Value
NLR	0.297 (0.138–0.642)	0.002
MLR	0.001 (0.001–0.240)	0.017
PLR	0.987 (0.977–0.998)	0.018
SII	0.996 (0.994–0.999)	0.002

All multivariable logistic regression analyses were tested separately for each ratio of interest (the NLR, MLR, PLR, and SII) and adjusted for covariates of age, sex, C-reactive protein, and disease duration. NLR = neutrophil-to-lymphocyte ratio; MLR = monocyte-to-lymphocyte ratio; PLR = platelet-to-lymphocyte ratio; SII = systemic immune-inflammation index; OR = odd ratio; and CI = confidence interval.

## Data Availability

Raw data from this study are available from the corresponding author upon reasonable request.

## Supplementary Materials

The following supporting information can be downloaded at: https://www.mdpi.com/article/10.3390/diagnostics14100992/s1, Table S1: Comparison of peripheral blood counts and ratios in different stages of NPDR. Table S2: Correlation between change in CMT following bevacizumab therapy and selected biomarkers.
