# Supplementary material for: The Predictors of Early Treatment Effectiveness of Intravitreal Bevacizumab Application in Patients with Diabetic Macular Edema
Source: Diagnostics (Basel). 2024 May 10;14(10):992. doi: 10.3390/diagnostics14100992 (PMC11120272; doi:10.3390/diagnostics14100992)
Supplement: Supplementary file 1 [file diagnostics-14-00992-s001.zip › diagnostics-2971281-supplementary.pdf]

Supplementary files

Supplementary Table S1. Comparison of peripheral blood counts and ratios in different stages of NPDR.

Variable	Diabetic retinopathy severity		
	**Moderate NPDR**	**Severe NPDR**	***P*-value**
HDL-C/apoB	1.54 (1.17-1.98)	1.10 (0.89-1.60)	0.012
Monocyte/ApoA-I	0.32 (0.25-0.39)	0.36 (0.27-0.43)	0.243
NLR	2.45 ± 1.05	2.26 ± 0.73	0.465
MLR	0.25 ± 0.09	0.27 ± 0.10	0.334
PLR	124.1 ± 47.5	112.9 ± 58.0	0.396
SII	579.7 ± 290.8	492.5 ± 262.1	0.240

Values are presented as median (interquartile range). NPDR = nonproliferative diabetic retinopathy; ApoB/ApoA-I = apolipoprotein B to apolipoprotein A-I ratio; HDL-C/apoB = high-density lipoprotein cholesterol-to-apolipoprotein B ratio; Monocyte/ApoA-I = monocyte-to-apolipoprotein A-I ratio; NLR = neutrophil-to-lymphocyte ratio; MLR = monocyte-to-lymphocyte ratio; PLR = platelet-to-lymphocyte ratio; SII = systemic immune-inflammation index.

Supplementary Table S2. Correlation between change in CMT following bevacizumab therapy and selected biomarkers.

**Variable**	**r-correlation coefficient***	***P*-value**
Baseline BCVA (logMAR)	-0.224	0.031
Baseline CMT	-0.310	0.001
Duration of DM	0.072	0.530
Baseline HbA1c	-0.080	0.489
Vitamin D	0.105	0.288
ApoB/ApoA-I	0.054	0.586
HDL/apoB	-0.083	0.466
Monocyte/ApoA-I	0.173	0.130

* Spearman's rank correlation coefficient BCVA = best-corrected visual acuity, CMT = central macular thickness; DM = diabetes mellitus; HbA1c = Haemoglobin A1c; ApoB/ApoA-I = apolipoprotein B to apolipoprotein A-I ratio; HDL/apoB = high-density lipoprotein cholesterol-to-apolipoprotein B ratio; Monocyte/ApoA-I = monocyte-to-apolipoprotein A-I ratio.
